# Navigating space and the developing mind

**DOI:** 10.3389/fpsyg.2025.1521487

**Published:** 2025-05-14

**Authors:** Adrienn Réka Németh, Sandra Stojić, Zoltan Nadasdy

**Affiliations:** ^1^Doctoral School of Psychology, ELTE Eötvös Loránd University, Budapest, Hungary; ^2^Institute of Psychology, ELTE Eötvös Loránd University, Budapest, Hungary; ^3^Department of Neurology, Dell School of Medicine, University of Texas at Austin, Austin, TX, United States; ^4^Zeto, Inc., Santa Clara, CA, United States

**Keywords:** spatial navigation, allocentric, egocentric, path integration, aging, development, theory of mind

## Abstract

In this article, we review the extensive and complex fabric of literature concerning the ontogenesis of spatial representations from earliest childhood to the elderly, including normal and abnormal aging (dementia and Alzheimer’s disease). We also revisit fundamental concepts of the neuronal representations of space, egocentric vs. allocentric reference frames, and path integration. We highlight a thread of contradictions in spatial cognition from infant cognition to the first breakthrough at around the age of four. The contradictions reemerge in the literature on age-related decline in spatial cognition. We argue that these contradictions derive from the incorrect assumption that path integration is exclusively associated with allocentric frames of references, hence, signatures of path integration are often taken as evidence for allocentric perspective-taking. We posit that several contradictions in the literature can be resolved by acknowledging that path integration is agnostic to the type of reference frame and can be implemented in both egocentric and allocentric frames of reference. By freeing the frames of reference from path integration, we arrive at a developmental trajectory consistent across cognitive development studies, enabling us to ask questions that may dissolve the obscurity of this topic. The new model also sheds light on the very early stage of spatial cognition.

## 1 Introduction: the concept of space

Together, space and time constitute the principal dimensions of our consciousness. They provide a framework to organize our memory, perception, and plans (Buzsáki and Llinás, 2017; Herweg and Kahana, 2018). Space without time is the arena of our interactions with the outside world. It is the coordinate system for all possible locations objects can take, including ourselves. Cognitive map and its neuronal representation in the brain determine an individual’s strategy of navigation (Ulanovsky, 2011). Therefore, the ability to navigate is inseparable from the nature of this cognitive space. The philosophical question of whether the representation of the space is *a priori* (Kant, 1998, as cited in Guyer and Wood, 1998) or the result of development (Clark, 1973; Lee and Spelke, 2010) is long overdue. The cultural history of the concept of space as an independent dimension of the world has faded in obscurity, and the earliest drawings and cave art inform us nothing about the difference between prehistoric and modern conceptualization of cognitive space (Keller, 2016).

There are two fundamental questions related to the *a priori* concept of space. First is the dimensionality of space, and second is the question of absolute vs. relative space. Concerning the first question, we know that vertebrates sense acceleration during locomotion and head-turns with their three semicircular arches in 3 more or less orthogonal planes (Berlin et al., 2013). While the 3-dimensional sampling of acceleration is consistent with the concept of the Cartesian coordinate system (Pérez, 2009), physics still owns us a clear answer about the dimensionality of space (Tegmark, 1997), and so does psychophysics; why do we perceive three?

Concerning the second question, it has been long debated whether space is a system of objects in relation to each other or only an abstract coordinate system. Western philosophers, from Aristotle to Descartes and Leibnitz, articulated the relational nature of space and motion. Although Leibnitz explicitly criticized Newton’s concept of absolute space and time, he did not provide a consistent alternative (Rovelli, 2006; Vailati, 1997). Despite its critics, the ubiquitous concept of 3-dimensional Cartesian space provided an elegant framework for Newton’s notion of absolute space (Stachel, 2005). Such a reference frame, independent of objects, motion, and the observer, is also a very accommodating notion as a cognitive frame of reference for navigation, specifically as an allocentric frame of reference. Whether the Newtonian concept of absolute space shaped our concept of space or it just happens to be coalescent with our intuition of a virtually infinite coordinate system encompassing all coexisting objects and events, we do not know. The cultural-historical origin of this concept is beyond our grasp. However, regarding the ontogenesis of this concept, we know with certainty that the notion of absolute space is the result of the developmental processes (Piaget and Inhelder, 1956; Lee and Spelke, 2010) and when combined with the concept of absolute time, it serves the main organizing dimensions of the episodic and autobiographic memory (Rice and Rubin, 2011).

Moreover, the concept of absolute space may extend beyond space itself. Numerous studies published over recent years argue that abstract semantical-conceptual representations in our brain are extensions of the spatial navigation system. According to this view (Epstein et al., 2017), spatial codes specific to the hippocampus and entorhinal cortex may be applied to non-spatial domains in the human brain and organize concepts in semantical spaces similar to cognitive maps, including social conceptual spaces. A signature of the putative spatial code was identified as a hexa-directional modulation of the BOLD response of fMRI, but direct single-unit electrophysiology proof is still lacking (Constantinescu et al., 2016; Mack et al., 2016).

In the following few, we aim to overview the literature on the development and decline of spatial cognition, with special regard to the egocentric and allocentric reference frames.

## 2 Basic concepts

The concept of cognitive map, introduced by Tolman (1948), provides the basis of our understanding of mammalian spatial navigation. Representations of spatial relations are results of learning and are stored in long-term memory (Madl et al., 2016). During spatial navigation, we rely on information registered in relation to two types of reference frames. One is an egocentric reference frame using a body-centered coordinate system. The other is an allocentric-type reference frame, which uses coordinate systems attached to the environment or other stable landmarks. Information acquired through our sensory organs is registered in egocentric reference frames. Egocentric reference frames can also be divided into retinotopic (eye-centered, also called gaze-centered), head-centered, and body-centered reference frames (Andersen et al., 1993; Klatzky, 1998). In contrast, objects can be represented in allocentric reference frames, such as the boundaries of the environment or architectural landmarks defined by the spatial relationship of objects with a relative viewpoint independence (Klatzky, 1998; Burgess, 2006). Typically, egocentric object representations are converted into allocentric representations during the construction phase of the cognitive map. However, the opposite may also happen when we navigate based on a cognitive map (using a graphical map, for instance), and we convert the allocentric into egocentric to compare the egocentric prediction to the actual egocentric sensory input. Consequently, we rely on both the egocentric and allocentric frames of reference during spatial navigation (Moffat, 2009).

What makes one spatial reference frame preferred over the other? While both ego and allocentric enable navigation and are supported by various viewpoints, the reference frames and viewpoints are not independent. Török et al. (2014) found a viewpoint-dependent bias in the effective use of spatial reference frames for navigation. Accordingly, by testing human subjects in virtual environments, certain viewpoints make the navigation relative to one reference frame more efficient than relative to another reference frame. Ground-level viewpoints, for instance, are more efficient in egocentric navigation in terms of time and distance to target than bird-eye views. Conversely, bird-eye views improve allocentric navigation performance relative to egocentric navigation. Hence, the efficacies of egocentric and allocentric reference frames for spatial navigation are not equivalent.

This relates to a potential source of confusion, namely, egocentric reference frames are often illustrated from a third-party point of view or from above. This may partly be due to the didactic habit of taking an outsider’s point of view when explaining spatial references, but it is misleading. Other sources correctly recognized that egocentric reference frames adhere primarily to a first-person point of view (for example, Ekstrom et al., 2014). While one can convert egocentric angular space to a bird’s-eye view map, the egocentric view is inherently expressed in polar coordinates from a first-person point of view (Wolbers and Wiener, 2014), with the observer in the center. In contrast, the allocentric reference frame is more likely expressed from an outside 3^rd^ party point of view.

Furthermore, the egocentric and allocentric spatial layouts support spatial retrieval differently. Serino et al. (2024) indicated better spatial memory performance of bodily stimuli in the egocentric recall task than in the allocentric one. This can be explained by the relationship between spatial representation and sensorimotor processing, which will be discussed later. It also seems that affection (e.g., positive aesthetic experience) is associated with egocentric memory due to its body-referenced nature (Babo-Rebelo et al., 2022).

According to Wang and Spelke (2002), humans primarily rely on dynamic and egocentric representations during navigation. For instance, the receptive field of a neuron is always anchored to a certain spatial point of reference. When the eyes make a saccade, the projections of objects shift relative to the center of the retina. Hence, the retinotopic projections of objects change with every saccade. Nevertheless, our visual cortex keeps track of these changes and subtracts them from the object coordinates. Hence, it is able to align the reference frame to these objects (Steinberg et al., 2022). If objects are stationary, this coordinate system provides an allocentric reference frame. By subtracting the vectors/tensors of position changes due to eye, head, and body movements, the brain progressively eliminates the effects of these accidental jitters while generating an observer- and viewpoint-independent representation of the environment. We can illustrate this idea with the concept of head-direction cells. According to Filimon (2015), the allocentric reference frame is a derivative of the egocentric frame of reference by applying a mental rotation through the relocation of the observer’s body. Therefore, argues Filimon, the allocentric reference will never be completely independent of the up-down, left–right dimensions defined by our body axis.

When discussing fundamental frames of reference, we must mention Siegel and White (1975) model, which is primarily a cognitive developmental model, later generalized by Montello (1998) into a framework applicable to the differentiation of spatial learning generally. According to the original model (Siegel and White, 1975), spatial navigation develops in stages. First, we memorize and recognize landmarks, which are salient objects and perceptual patterns in the environment that do not contain metric information. Route knowledge is created by connecting landmarks into a sequential order through movement. During this process, we form expectations about potentially occurring landmarks and the decisions associated with them. This type of knowledge is egocentric (Shelton and McNamara, 2001; König et al., 2019). The most complex level of spatial learning is survey knowledge, which is a metric, map-like representation of the environment. It includes the network of routes and landmarks as well. According to Siegel and White (1975), the use of shortcuts, the creation of new routes, and the ability to determine the direction of landmarks are evidence of this allocentric-based spatial knowledge (Montello, 1998; König et al., 2019). Montello (1998) critique suggests that spatial knowledge does not develop in a strictly hierarchical manner, assuming map-like knowledge from the beginning.

Concerning human spatial navigation Wang and Spelke (2002) identified three fundamental systems: (i) Path integration, which dynamically updates our current position relative to significant environmental locations. (ii) View-dependent place recognition, which involves matching the visual field to viewpoint-dependent representations of landmarks. (iii) Reorientation, which complements path integration when it fails and helps the navigator restore their position.

Path integration (PI) enables us to determine our position based on proprioception, optic flow, and vestibular input, collectively referred to as idiothetic cues, as opposed to the allothetic (or allocentric) cues discussed above (Loomis et al., 1999). PI is the method by which the navigator stores and aggregates recent traces of straight path segments starting with the initial position where the vectorial sum of these segments represents the actual position. Hence, the navigator can always localize itself by updating the vectorial sum and finding shortcuts to returning to the starting point (Collett and Collett, 2000). Regarding gender differences in PI, Coutrot et al. (2019) did not find significant differences between male and female subjects, and this concordance is maintained throughout the lifespan (Yu et al., 2021).

Because PI enables shortcuts and PI contributes to the formation of cognitive maps, shortcuts are often mistakenly taken as evidence for cognitive maps. However, the use of PI in relation to allocentric and egocentric navigation is still controversial, which we present in the following paragraphs.

## 3 The neuronal background of the spatial navigation

The neuronal underpinning of spatial navigation has been extensively studied over the last 50 years. Converging human neuroimaging and animal electrophysiology unraveled the role of the medial temporal lobe and, in particular, the hippocampus in spatial navigation (Antonova et al., 2009; Brown and Chrastil, 2019; Li and King, 2019; Rinaldi et al., 2020). According to neuroimaging, the parietal cortex and the hippocampus are part of the episodic memory system (Dickerson and Eichenbaum, 2010), which integrates semantic content into sequentially organized episodic memories (Buzsáki and Llinás, 2017). The right parietal cortical areas are implicated in egocentric strategies (Maguire et al., 1998). The parietal lobe contributes to egocentric orientation through the execution of multiple psychological functions, including spatial attention (Luria, 1973; Andersen and Buneo, 2002; Souza-Couto et al., 2023), spatial awareness, multisensory integration (Andersen and Buneo, 2002), and somatosensory processing (Navon, 1997; Souza-Couto et al., 2023). Likewise, specific areas in the dorsal striatum, such as the caudate, are responsible for egocentrically defined motor movements (Wolbers and Wiener, 2014). The hippocampus and the parahippocampal areas are involved with allocentric navigation (Maguire et al., 1998). The hippocampus, as a core component of the long-term episodic memory system, maintains and constructs world-centered perceptual information called the cognitive map and provides flexible navigation in space (O'Keefe and Nadel, 1978; Bird and Burgess, 2008).

Concerning the role of the mesial temporal lobe, three types of cells play key roles in spatial navigation and cognitive map formation: the place cells (O'Keefe and Dostrovsky, 1971), the grid cells (Hafting et al., 2005; Moser et al., 2008) and the head direction cells (Taube et al., 1990). In addition, we distinguish border cells (Solstad et al., 2008; Savelli et al., 2008), border-vector cells (Barry et al., 2006), and conjunctive cells (Sargolini et al., 2006), which encode the combination of the head-direction and the place information. Place cells are located in the CA1, CA2, and CA3 areas of the hippocampus and are activated when the animal traverses an allocentrically defined location (O’Keefe, 1976), regardless of the direction and time of transit. Furthermore, place cells are tuned to distances relative to the environmental boundaries, rather than to local cues attached to the place. Additionally, these cells integrate self-motion information via neuronal input from the entorhinal cortex, which updates the navigator’s perceived location (Bird and Burgess, 2008). Hippocampus may play a key role in PI as well (Bicanski and Burgess, 2020). In contrast, grid cells of the medial entorhinal cortex show allocentric activity in multiple locations that define a uniform equidistant triangular grid that forms a hexagonal tessellation pattern (Hafting et al., 2005). According to Wolbers and Wiener (2014), these grid cells may also play a role in PI. They contribute to the construction of a viewpoint-independent frame of reference. The head-direction cells in the subiculum, the striatum, and the thalamus complement the role of place cells and grid cells, all of which were originally described in rodents. The head-direction cells encode the angle of the head relative to an allocentric radial coordinate system regardless of the orientation of the body. These three cell types collectively provide the fundamental components of spatial navigation (Hafting et al., 2005).

Place cells, grid cells and head direction cells are considered to be part of the allocentric system. They are activated by the individual being at that location or, crossing that location or heading in that direction. However, a subset of place cells respond to other individuals being at the specific location. These so called “social place cells” may play an important role in dissociating the egocentric frame of references from allocentric during the cognitive development in children that enables them to solve the false belief tasks in the context of “theory-of-mind” (see in 5.1 below) (Omer et al., 2018; Danjo et al., 2018).

If these cell types contribute to spatial navigation and represent a significant volume of the mesial temporal lobe, one might expect adaptative changes in morphology. In their seminal study, Maguire et al. (2000) studied the role of such adaptation to the environment in a navigation experiment with the participation of London taxi drivers and showed that they demonstrated structural enhancement in the posterior and anterior areas of the hippocampus by using MRI which was explained with their profession. The alteration of the grey matter of the hippocampus correlated with the years of experience. Although later studies (Weisberg et al., 2019; Weisberg and Ekstrom, 2021) indicate that the relationship between hippocampal volume and navigational behavior is only confirmed in extreme cases, such as dementia or expert navigators like taxi drivers. In normal cases, this brain plasticity does not cause significant structural changes that would appear in brain volume. Alternatively, the neuronal changes accompanying navigational behavior are not so much due to changes in hippocampal volume, but rather to the reconfiguration of vascular and ganglionic resources in response to environmental changes.

Finally, to conclude the neuroanatomy section, we elaborate briefly a relevant model and a framework helping to elucidate the neural basis of spatial memory. According to a model by Byrne et al. (2007) (also referred to as the BBB model), events are stored alongside spatial context, and this spatial context plays a fundamental role in both encoding and retrieval processes. The hippocampus and parahippocampal cortex create allocentric maps that encode spatial context, such as landmarks, distances, and directions. For example, a memory of an event includes the geometry of the room where the event occurred. During retrieval, this contextual spatial information is reconstructed through pattern completion, a process that fills in missing details. Based on the BBB model, spatial memory and visual imagination share a common neurological background. Byrne et al. (2007) explain the navigation process as follows: the hippocampus enables the retrieval of the allocentric map (Byrne et al., 2007), the parietal cortex transforms these into egocentric information (Burgess, 2006) and the prefrontal cortex simulates mental navigation (Addis et al., 2007).

Kravitz et al. (2011) challenged the previous models of visual processing pathways in the brain, traditionally divided into a ventral stream or “what” processing, and a dorsal stream or “where” processing (Ungerleider and Mishkin, 1982), providing a novel neural framework for visuospatial processing. Although the distinction of cortical visual processing into discrete dorsal and ventral streams has served as one of the fundamental frameworks within the field of visual neuroscience, it was not clear how the information conveyed by these two separated systems merges into the coherent visual percept. The team identified three distinct pathways branching out from the dorsal stream, i.e., projections to the prefrontal and premotor cortices and to the medial temporal lobe that support both conscious and non-conscious visuospatial processing, including navigation, visually guided actions, and spatial working memory (Kravitz et al., 2011). In their review, Kravitz et al. (2011) also suggested that, since both the ventral stream and the parietal temporal pathways project to MTL (Mishkin et al., 1997), the major point of perceptual integration likely occurs in the hippocampus. Within the proposed neural framework, it was assumed that the ventral stream supplies the parahippocampal cortex with the information necessary for representing landmarks, while the parietal-medial temporal pathway provides information about the spatial context that is essential for navigational purposes (Kravitz et al., 2011).

## 4 The measurements of the spatial navigation ability

According to the summary of Klencklen et al. (2012) and Pullano and Foti (2022), there are copious methods available to quantify spatial navigation, which can be classified based on different dimensions. Route knowledge, spatial memory, spatial manipulation, and spatial working memory can be explored according to the navigation process. We can distinguish according to the type of environment, investigating real or physical and virtual spaces. Based on these reviews, when selecting navigational measurement tools, it is important to identify the size of the space being studied. There are small spaces, which are up to room-sized, reaching space, which enables manipulation within arm’s length, and large-scale spaces. Montello (1993) defined four types of space according to their size: figural, vista, environmental, and geographical. Here, we have adopted Montello’s suggestion that figural and geographical spaces are either too small or too large to be discussed in detail from a navigation perspective. As Wolbers and Wiener (2014) pointed out, the distinction between vista space and environmental space is relevant to navigation. Vista spaces (e.g., rooms, gardens) can be visually perceived from a single vantage point without significant changes in position. Environmental spaces enroll buildings, neighborhoods, and cities, which can be conceived through locomotion. We use different components of egocentric navigation in these varying sizes and complexities of spaces. In vista spaces, a single response learning is sufficient, whereas in environmental space, associative elements are also important for egocentric navigation, such as turning right at a certain landmark. The execution of these elements takes longer than a single motor response in a vista space (Wolbers and Wiener, 2014).

Numerous spatial tasks have been developed to test allocentric reference frames. One is the Triangle Completion Task (Loomis et al., 1993), which is a typical device for measuring PI (Chrastil and Warren, 2008). Allocentric information processing can also be measured by map construction tasks and map-based positioning tasks (Klencklen et al., 2012). However, maps do not exclusively require allocentric references and can also be solved using egocentric strategies (e.g., Mittelstaedt and Mittelstaedt, 1982; Bennett, 1996) – a distinction we elaborate on in a later paragraph.

Another is the Morris Water Maze task (MWM) (Morris et al., 1982), which became the most widely used tool to test spatial learning and the role of external cues in allocentric spatial navigation (Klencklen et al., 2012). This experimental setup can easily be replicated in virtual reality since the environment can flexibly be updated by software without the participant changing his/her real physical position. As a result, VR introduces a conflict between egocentric and allocentric cues by updating the allocentric while leaving the egocentric cues (such as proprioception) unchanged. This conflict between the allocentric and egocentric strategies in virtual environments complicates the interpretation of results (Moffat, 2009; Diersch and Wolbers, 2019). Another concern with the MWM task was presented by Wolbers and Wiener (2014) and Burgess et al. (2002). They argued that the MWM can be solved by computing the allocentric vectors between the outer landmarks and the platform. Specifically, the navigation in the maze does not require the organism to know its own location, which can be calculated based on the landmarks. Likewise, the various split maze tasks (e.g., T-maze, Y-maze) are also affected by the lack of self-location (Wolbers and Wiener, 2014).

### 4.1 Methodological and conceptual problems in the research of spatial navigation

At this point, we wish to highlight two key inconsistencies in the literature concerning path integration (PI), which may partly account for the contradicting findings regarding age-related changes in spatial cognition. One such inconsistency involves the frequent conflation of PI with the use of allocentric reference frames. This reflects a broader methodological issue present in many spatial navigation tasks that include visual cues: the position of the target relative to the navigator can often be computed using solely egocentric information derived from these cues (Wolbers and Wiener, 2014; Markus et al., 1995; Burgess et al., 2002). Although these tasks are designed to engage allocentric reference frames, they can often be solved using egocentric strategies. We explore this issue in greater detail in the following section. Another inconsistency lies in the assumption that the cognitive map is a direct product of path integration (PI). It is often suggested in the literature that tasks requiring PI inherently lead to the formation of a cognitive map (Samsonovich and McNaughton, 1997; Jayakumar et al., 2019; Etienne and Jeffery, 2004; McNaughton et al., 1996; O’Keefe, 1976; Redish and Touretzky, 1997). Indeed, several computational models of the hippocampus are based on the premise that it functions as a path integrator (McNaughton et al., 1991). However, this interpretation blurs the conceptual boundary between PI and the use of allocentric reference frames. Savelli and Knierim (2019) have explicitly addressed the need to disentangle these constructs. Moreover, earlier empirical studies have questioned the necessity of allocentric representations for successful path integration (Gothard et al., 2001; Save et al., 1998), suggesting that PI can operate independently of a cognitive map or global spatial framework.

#### 4.1.1 Critic of path integration implying allocentric frame of reference

This paragraph concerns the confusing relationship between PI and allocentric frame of reference. It is agreed that PI primarily relies on idiothetic (self-motion) signals deriving from vestibular, proprioceptive, visual flow, and motor sources (Buzsáki and Moser, 2013; Etienne and Jeffery, 2004; Redish, 1999). More recently, human studies added optic flow to the egocentric cues (Bierbrauer et al., 2020; Howett et al., 2019), and studies on congenitally blind people reinforced the predominance of egocentric cues in PI (Corazzini et al., 2010). While PI is defined relative to egocentrically defined targets, it can contribute to the construction of allocentric maps. However, that contribution is still hypothetical. Several models have attempted to relate PI to grid cell activity (McNaughton et al., 2006; Fiete et al., 2008; Gil et al., 2018; Bush et al., 2015). Because grid cells provide an allocentric coordinate system for spatial navigation, they naturally impose an allocentric frame of reference on PI. This generated a conceptual drift of PI from egocentric to allocentric navigation that led to the tacit assumption that PI involves an allocentric frame of reference.

One way to reconcile the inconsistent relationship between PI and reference frames is to assume that while PI primarily derives from idiothetic cues, it can also operate on both egocentric and allocentric reference frames. Given the allocentric coordinates of the agent and targets, PI can compute shortcuts based on allocentric cues, but that is different from egocentric PI, even though the outcomes are the same. At this point, however, we do not know if allocentric PI is necessary for allocentric navigation on cognitive maps. Until this link between PI and allocentric representations is empirically proven, PI should not be considered as a primary attribute of allocentric navigation because it can be computed relative to egocentric reference frames. Therefore, it is a mistake to infer an allocentric map and, furthermore, a cognitive map from PI as we often see it in the literature (for instance: Menzel et al., 2005; Blaser et al., 2013; Wang, 2016). To illustrate this, let us take a closer look at the issue in the context of egocentric and allocentric navigation.

**Figure 1:**
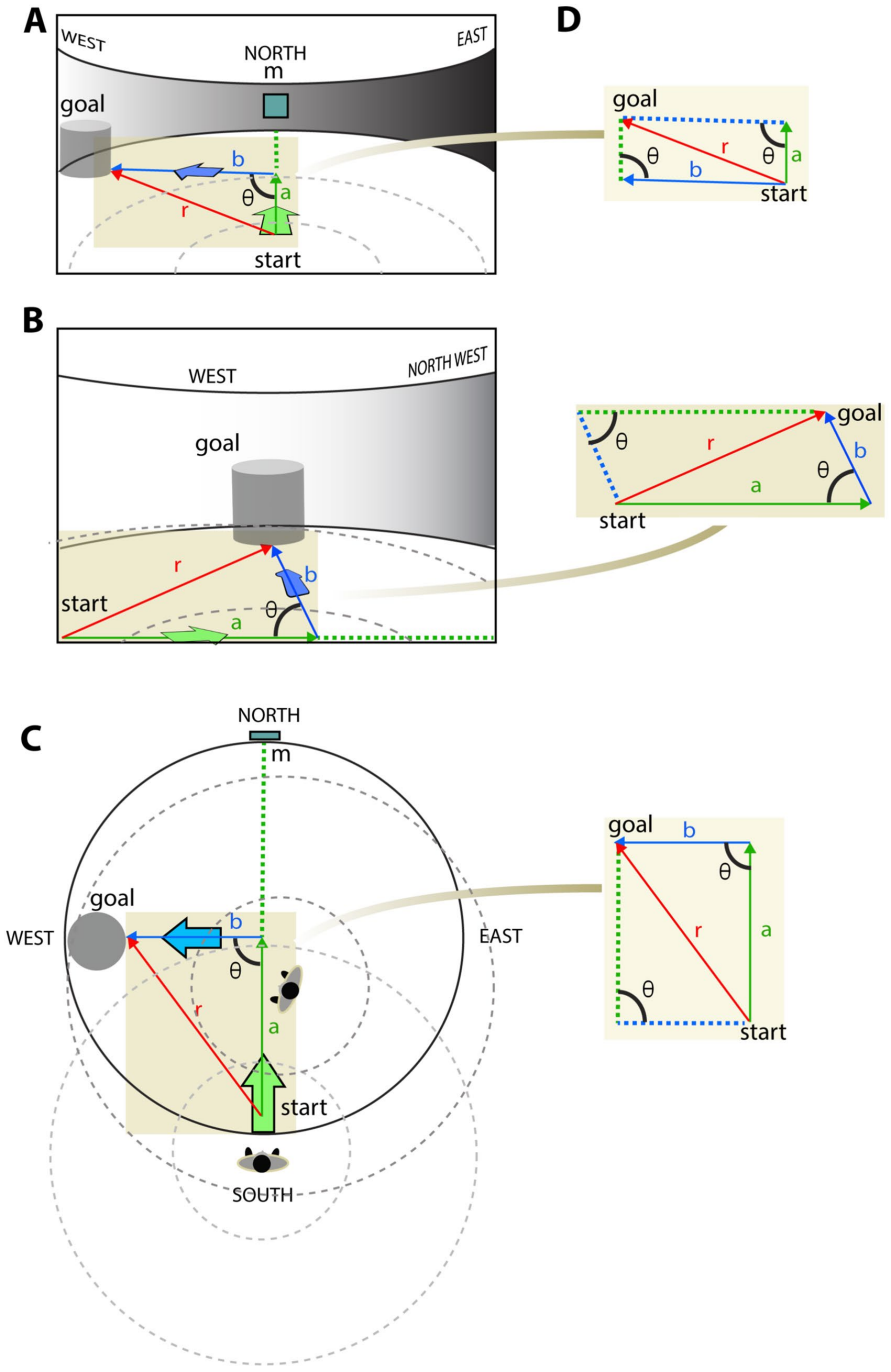
Equivalence of egocentric vs. allocentric path integration. Given a circular arena, an observer plans to approach a cylindrical shape object (goal) at the edge of the arena from a starting point (start) along two straight lines (a and b are marked by green and blue colors, respectively) with a *θ*-degree turn between them. **(A)** The described movement is visually represented from an egocentric first-person point of view at start. Red arrow (r) represents the resultant vector *r^2^ = a^2^ + b^2^ + 2ab cosθ*, where *θ* denotes the angle between the two movement vectors (*a* and *b*) as they project on the observer’s screen. **(B)** An updated view after moving the observer along the green path toward the turning point. As the viewpoint moves, the *a, b,* and *r* vectors change their length and *θ* angle. **(C)** The same pathway is represented from a map-like allocentric point of view with path integration. While both egocentric **(A,B)** and allocentric **(C)** path integrations provide correct estimates of the goal coordinates within their coordinate systems, the length of vectors and their angles differ between reference frames. **(D)** The corresponding vector summation extracted from the different point of views. While the difference between projections of the same vector summation is discernable they all correctly estimate the shortcut between the objects.

Consider a task that involves PI in a circular arena to model the navigation scenarios under egocentric and allocentric conditions (Figure 1). For simplicity, we render the target cylinder visible. No matter where the navigator starts and what additional target it has to pass, the landmarks precisely define the shortest path between the origin and the target. Once the target position is acquired relative to the landmarks, the navigator is able to maintain its direction (bearing) toward the target during navigation. Let us look at the PI from an egocentric (first-person) point of view and an allocentric (bird-eye) point of view (Figures 1A–C). The figure illustrates that despite the difference in angles and component vectors between the egocentric and allocentric projections, the resultant vector defines the shortest path between the start and goal equally precisely under the two conditions. Since PI can be solved by allocentric and egocentric navigation, as noted in other studies (e.g., Wolbers and Wiener, 2014; Ekstrom et al., 2014), it is not necessary to assume allocentric representation to explain it. The equivalence of the allocentric and egocentric vector summation is evident under such a static scenario, as depicted in Figures 1A,C. However, it gets more complicated when the observer, and likewise the viewpoint, is moving Figure 1B.

To disentangle the relationship between egocentric and allocentric reference frames with regard to PI during movement, we need to introduce two types of coordinate systems, Cartesian and polar (or spherical), and a class of transformations called homography.

A static point in the allocentric space is defined by its Cartesian coordinates x, y, and z. The same point in an egocentric coordinate system is described by three polar coordinates: radial distance *r* relative to the observer as origin, polar angle *θ* relative to the z-axis, and azimuthal angle *ϕ* in the xy-plane from the x-axis. Without motion, given the x, y and z coordinates of a point in 3D space, the equivalence of egocentric and allocentric reference frames with respect to PI is provided by the conversion between them.

To compute the radial distance *d*:


$$d=\sqrt{x^{2}+y^{2}+z^{2}},$$

the polar angle *θ* (Colatitude):


$$\theta =\cos^{-1}(\frac{z}{r}),$$

and azimuthal angle ϕ (Longitude):


$$\phi =\tan^{-1}(\frac{y}{x}).$$

Modeling the PI in a moving coordinate system requires homography when the observer is moving or the object is moving or both. Homography is a projective transformation between two planes or a mapping between two planar projections of the same object(s). Homography can also be applied to recover the relative position of the observer when the observer or the observed objects are moving. Concerning the transformations between egocentric and allocentric reference frames, we need to distinguish two uses of homography: (i) one is to describe the relationship between two images of the same scene taken from different perspectives, and (ii) as a projective geometry that maps the original 3D scene represented in an orthogonal Cartesian space and transforms it to a 2D surface from the observer’s viewpoint. While the first use case of homography can be solved by linear or affine transformations between egocentric polar coordinates (Beusmans, 1993; Schuchert and Scharr, 2009; Fleet and Weiss, 2006), the second class involves fundamentally nonlinear transformations.

Regarding PI, computing shortcuts in allocentric space requires vector summation. The motion does not complicate this because it only involves updating the positions of the observer in the environment. Computing shortcuts in egocentric space during motion requires affine transformations between projections of the same object in the observer’s polar coordinate system, which is also a simple matrix operation. This can be done without relating the projections to the 3D Cartesian coordinates of the objects, i.e., the structure of the world that generated it. Hence, solving the shortcut problem in navigation with PI does not require nonlinear transformation because we do not need to recover the original 3D cartesian coordinates of the objects, i.e., solving the inverse problem. In other words, the subject does not need to build an allocentric representation of objects to compute shortcuts.

Just because the two tasks of solving the inverse problem and updating the PI in egocentric coordinates during motion are mathematically independent does not mean they must be biologically independent, either. It is conceivable that a deep-learning-type multilayer neuronal network using a transformer can solve both problems at once.

In summary, (i) a vectorial sum of component vectors projecting to the retina representing an egocentric first-person viewpoint provides shortcuts to the target before moving, and (ii) that shortcut is equivalent to the solution obtained by vectorial summation of allocentric component vectors regarded from an abstract top-down or bird-eye view (Figure 1). Hence, computing the resultant vector r is the same in both allocentric and egocentric coordinate systems:


$$r^{2}=a^{2}+b^{2}+2\mathrm{ab}\;\cos\;\theta ,$$

Where *a* and *b* are the lengths of the two-component vectors and *θ* is the angle between them.

PI is generally agnostic to the sensory modality as long as the sensors inform about the target positions, such as binaural sound localization or odor localization based on the spatial gradient of odorant molecules. Nevertheless, different sensory inputs may contribute differentially to egocentric vs. allocentric reference frames. Since the pioneering work of Knudsen and Konishi (1978), we know that auditory space maps on the inferior colliculus of the owl provide an egocentric soundscape of the environment.

Vision conveys a primarily egocentric sensory input, and results show the performance decline in rodents when deprived of being able to look around (Arolfo et al., 1994). Therefore, the contribution of visual egocentric cues to navigation is beyond doubt when available. The MWM task can also be solved by relying on visual matching (Markus et al., 1995; Burgess et al., 2002) with or without the allocentric frame of reference (Wolbers and Wiener, 2014). Consistent with this notion, the MWM task can be solved by patients with hippocampal lesions, too (Shrager et al., 2008), whose parietal or retrosplenial areas supporting the egocentric reference frames are intact.

In addition, concerning the MWM task, whether the task requires using a cognitive map or procedural learning is not well-defined (Arolfo et al., 1994; Iaria et al., 2003). The activation of the hippocampus is ambiguous during the platform search. On the other hand, in the case of hippocampal injury, the hippocampus is not necessary for PI since the PI can be solved by areas closer to the sensory areas, such as the retrosplenial cortex (Chrastil et al., 2015). Instead, the hippocampus is implicated when the task involves memory to recall the location of the hidden platform and the former underwater navigation experience (Bird and Burgess, 2008).

Despite all these methodological limitations, the literature generally references the MWM task as a suitable tool for measuring allocentric navigation (Wolbers and Wiener, 2014; Moffat, 2009).

#### 4.1.2 Critic of the shortcuts and the cognitive maps equivalence

The other misconception about spatial navigation dates back several decades to Tolman (1948). Tolman (1948) interpreted the shortcuts in his experiment with the rodents as evidence of the cognitive map. O'Keefe and Nadel (1978) also refer to the shortcuts as a cornerstone of the cognitive maps (O'Keefe and Nadel, 1978 pp. 68):

“Peters (1973) has described hunting behavior in wolf-packs which, he feels, necessitates the use of the cognitive mapping concept. He cites three pieces of evidence: (1) wolves can take intentional shortcuts or detours; (2) packs can split up and re-group at some distant point, beyond the effective range of howling, such that some idea of distance and direction is required; (3) wolves can return to a rendezvous point where pups have been left *from any direction.* All this strongly implies a map-like organization of their psychological space. (…)”

Tolman’s interpretation of shortcuts as a hallmark of “cognitive maps” had a large impact on advancing the field beyond behaviorism, especially among those who were seeking evidence of neuronal representations in the brain beyond conditioned reflexes. The idea of cognitive maps inspired new experiments, including the groundbreaking study by O’Keefe and Nadel that led to the discovery of place cells, foundational for the entire field of spatial cognition. Retrospectively, it should not discount Tolman’s merit that his experimental finding, in rigorous terms, did not prove the existence of the cognitive map, nor was it reproducible on human subjects (Wilson and Wilson, 2018). The discovery of place cells was proof that neurons form abstract representations in the brain that could not be derived by transformation of sensory input but rather a derivative of aggregate experience. Unlike the invariances of colors, shapes, sizes, surface textures, or acoustic patterns extracted from sensory input, the invariances of locations are fundamentally different.

Since Tolman’s experiment, the ability to take shortcuts has been proven in invertebrates (Gould, 1986; Menzel et al., 1998; Müller and Wehner, 1988). In the experiment of Menzel et al. (2005), honeybees were able to navigate relative to a landmark and find the shortest route to the hive. Owing to Tolman’s inductive reasoning, the concept of cognitive maps was extended to insects despite the absence of the hippocampus, the key brain structure in mammals. Since Tolman, many other authors (Arleo and Rondi-Reig, 2007; Klencklen et al., 2012) consider shortcuts as evidence of the allocentric frame of reference and the cognitive map. Using PI for navigation has been reported on various classes of insects (Menzel et al., 2005) and other invertebrates in general (Collett, 2003), and amphibians (Fischer et al., 2001) that they routinely use shortcuts during their navigation without the hippocampus. Whether these species rely on complex olfactory clues, proprioception, polarized light, and directional force fields, such as the earth’s magnetic field, or a combination of all those is not always conclusive. According to Bennett (1996), experiments done on spatial navigation of various species claiming the prevalence of cognitive maps do not necessarily address the question of whether or not the tasks can be solved purely by PI. Moreover, these studies could not rule out that the shortcuts observed were computed based on PI without a cognitive map formation.

A recent study has found that insects use egocentric vectors stored in long-term memory to navigate that enable them to high-level navigation without a cognitive map (Patel et al., 2024). Cruse and Wehner (2011) modeled the map-like behavior of the insects using an artificial neuronal network. According to their results, the former outcomes of the insect’s experiments can be explained by PI and route knowledge without assuming a coherent 2D map. Likewise, combined experimental, anatomical, and modeling studies revealed that the central complex in the insect nervous system implements PI by an elegant circuitry (Stone et al., 2017). According to their model, the inputs from compass neurons utilizing polarized-light and speed-encoding neurons relying on optic-flow converging on the central complex neurons enable ring-attractor dynamics, which can explain the remarkable precision of PI and enable reliable navigation.

The critical review of this body of literature raises two important questions: is the hippocampus necessary for the formation of a cognitive map? Also, are cognitive maps necessarily allocentric? Regarding the first question, insect studies suggest the hippocampus is not necessary (Menzel et al., 2005; Cruse and Wehner, 2011), but rodent studies argue otherwise (O'Keefe and Nadel, 1978). While the association between the hippocampus and cognitive map formation is predominant, the dependency of cognitive maps on the hippocampus was called into question (Eichenbaum, 2017; Shrager et al., 2008).

This contradiction can be solved by considering that although PI may lead to shortcuts, it does not imply using an allocentric reference frame or depending on a cognitive map. It is conceivable that PI with, its most basic function, such as leaving the nest and returning, is sufficient for numerous solutions of shortcut-like behavior (Mittelstaedt and Mittelstaedt, 1982).

The dissociation between shortcut-behavior and cognitive-map-based navigation is also evident in human spatial memory. A series of studies (e.g., Teng and Squire, 1999) demonstrated this distinction by testing patients with bilateral medial temporal lobe lesions—including the hippocampus and hippocampal-entorhinal cortex—alongside healthy controls (Shrager et al., 2008). Participants were blindfolded and guided along a path, then asked to either point to or return to the starting location. Remarkably, patients with hippocampal damage performed comparably to controls on self-localization and shortcut tasks, as long as no long-term memory component was involved. However, when a delay and distractors were introduced before the test phase, patients exhibited significant impairments in path integration (PI) and self-localization. These findings suggest that the parietal lobe, which supports egocentric multimodal spatial representations, is sufficient for immediate PI in the absence of hippocampal function (Etienne and Jeffery, 2004; Mesulam, 1981; Save and Moghaddam, 1996). This contrasts with rodent models, in which both immediate and memory-dependent shortcuts depend on the integrity of the medial temporal lobe (Kim et al., 2013). Anatomically, this aligns with the proposed division between egocentric and allocentric PI: the former being primarily supported by the parietal cortex and the latter by the hippocampal–entorhinal system (Kim et al., 2015).

Regarding cognitive maps, it is important to note that the pointing task did not explicitly require participants to construct or visualize a cognitive map; subjects were not instructed to draw or conceptualize spatial layouts. Moreover, successful shortcut performance did not appear to depend on cognitive map-based navigation, nor did cognitive map formation depend on shortcuts and PI. However, the spatial memory component of the task strongly depended on the integrity of the medial temporal lobe. The fact that the hippocampus-dependent memory performance became resistant to distraction over time suggests that the cumulative process of consolidating the memory of paths leading to the target is dependent on the hippocampus and entorhinal cortex. These findings lend further support to the foundational hypothesis proposed by O'Keefe and Nadel (1978), which posits that the neural mechanisms underlying the construction of cognitive maps are defining features of the medial temporal lobe, particularly the hippocampal–entorhinal system in the mammalian brain (O'Keefe and Nadel, 1978).

#### 4.1.3 Fundamental notions and conceptual confusion

In addition to the methodological problems, the inconsistent use of the basic concepts may also contribute the conceptual confusion (Foley et al., 2015). Grush (2000) draws attention to the heterogeneity of the context in which the concept of allocentric is used. Sometimes, the literature uses the allocentric notion for egocentric situations in which we localize an object relative to other objects, but the localization relies on the difference in angle relative to the observer. Hence, it is egocentric. In this case, we should not use the term “allocentric reference.” There are many correct versions, too. For instance, when a viewpoint-independent object is at the center of the reference. Also, when the point of view is neutral, such as in the case of bird’s eye perspective and maps. Adding to the confusion, allocentric reference frame in philosophy, is also associated with the “view from nowhere,” reflecting the idea that subjectivity cannot be entirely removed from any perspective. The term *nemocentric* is sometimes used to denote true neutrality in this context. The author argues that objective representation is less about the absence of a specific point of view and more about the ability to adopt “any” possible point of view. He further refines the concept of “any” to refer to the ability to take on some possible point of view, linking the criterion of objectivity to the observer’s capacity to perceive independently of their own current perspective. This suggests that our ability to engage with alternative allocentric perspectives ensures that everything within the egocentric space remains accessible from different points of view (Grush, 2000).

In our definition, allocentric means the viewpoint-independent coordinates of an object. In other words, it is a coordinate system that allows virtual viewpoints to disengage from the observer’s own perspective. As a result, maps require an allocentric strategy, except for the bird-eye perspective, which permits to rely on either egocentric or allocentric reference frames. In order to distinguish between these two scenarios, we have to consider the sensory and motor aspects of the navigation. Switching the viewpoint from egocentric to allocentric does not necessarily change the reference to movement control. Consider computer games in which the player controls the avatar visually from a bird-eye perspective. The movement control of the avatar can be mapped egocentrically or allocentrically. In the egocentric case, when the player hits the ‘right’ key, the environment rotates clockwise, and the avatar will turn to the left relative to its body axis or, alternatively, the environment rotates anti-clockwise, and the avatar will turn to the right, depending on the game implementation. In contrast, in the allocentric control, the avatar, after hitting the ‘right’ key, will move toward the eastern edge of the screen (Török et al., 2014).

The question arises: if the allocentric representation requires so many transformations, what is the advantage of it? Switching to allocentric reference pays off in keeping track of the motion of the body. We posit, it is much easier to update the 2 or 3 coordinates of the position in a 2 or 3-D coordinate system fixed to the exterior environment than updating the optic flow (the sequence of views) during motion; each frame of the optic flow is obtained from an egocentric viewpoint while moving. Using an allocentric reference, we have to store the X, Y, and Z coordinates only to reconstruct the route, while in the egocentric frame of reference, we have to store a stack of 2D images to reproduce the optic flow as every pixel of those images changes with the agent’s position. A similar argument was put forward by Wolbers and Wiener (2014).

So far, we reviewed the diversity of theoretical and methodological approaches related to the complexity of spatial navigation. Considering the time course of ontogenetic development of navigation from the egocentric PI to a full-fledged cognitive map will clarify the terminology. Among the multiple developmental scenarios, it is conceivable that there is a specific human scenario for the development of spatial concepts. To draw the trajectory of the development of spatial concepts, we review the literature on the cognitive development of spatial concepts in the following paragraphs.

## 5 The spatial navigation ability of children

Spatial navigation requires multiple cognitive abilities, and it has a special developmental course from childhood to the elderly (Moffat, 2009; Bullens et al., 2010; Lester et al., 2017). We emphasize the role of the sensory input in the development of the navigation. For instance, visual inputs dominate human spatial navigation. A fully developed 3-dimensional vision enables the precise evaluation of the orientation and distance of a spatial reference point. In addition, the development of the visual system determines the availability of reference frames, and reference frames define the developmental milestones. It has been demonstrated that congenitally blind people have difficulty in allocentric navigation, especially at a larger scale environment requiring locomotion (Iachini et al., 2014) and the deficit in the shift from egocentric to allocentric representations (Ruggiero et al., 2018).

Despite the wealth of data collected in the context of children’s spatial cognition, many questions remained open (Bullens et al., 2010). Piaget and Inhelder (1956) proposed the model that egocentric reference frames predate allocentric before the age of seven. The initial preference for egocentric orientation is supported by extensive research (Huttenlocher and Presson, 1973; Acredolo, 1978; Rieser, 1979; van den Brink and Janzen, 2013; Bullens et al., 2010; Wang and Spelke, 2002; Vasilyeva and Lourenco, 2012; Fernandez-Baizan et al., 2021). According to this approach, viewpoint-independent strategies require matured spatial cognition. However, studies disagree on the onset time of allocentric reference frames, and several authors localize the use of allocentric strategies in time much earlier than Piaget (e.g., Acredolo, 1990; Rieser, 1979; Bullens et al., 2010). However, in many cases the early ‘allocentric data’ derives from navigation experiments orienting relative to landmarks. Contrary to this model, children are able to use primordial-allocentric representations at birth, but at a certain age and under experimental conditions, they prefer egocentric representations (Landau and Spelke, 1984; Kaufman and Needham, 1999; Newcombe, 2019). In an experiment by Kaufman and Needham (1999), 6-and-a-half-month-old children were able to encode the spatial changes allocentrically without the help of landmarks if the task was cognitively not challenging. This dishabituation task tested their ability to track the relative position of a goal-object. The egocentric preference may reflect the limitation of immature executive functions and is further dependent on the development of the hippocampus. The theory postulates that the predominance of the egocentric perspective is caused by children’s immature executive function, which makes them unable to handle competitive body-centered and environment-centered representations (Nardini et al., 2006). Nardini et al. (2009) raise another important question: whether children who correctly solve allocentric tasks also acquire comprehensive spatial knowledge, which a mental map requires, or if it merely involves mental operations that allow participants to adopt an independent viewpoint.

According to results using virtual reality implementation of spatial navigation, children at the age of 5 can navigate virtual Star Maze environments (which utilize visual cues similar to those in the MWM) that require both egocentric and allocentric strategies, but they only achieve the level equivalent to adults at the age of 10 (Bullens et al., 2010). Nardini et al. (2009) reported a similar result in a series of experiments conducted in a real-world environment, where visual cues and path integration information from movement were excluded as potential aids for allocentric strategies. While 4-year-olds were more efficient in egocentric viewpoint-dependent navigation, the ability to use allocentric landmarks was also apparent at this age. Similarly, 5-year-olds were found to be alternating between different strategies, but they only become confident in allocentric navigation and can decode their environment’s spatial structure by the age of 6–8. Van Hoogmoed et al. (2022) suggest that allocentric navigation based on proximal landmarks develops between the ages of 5 and 8 and becomes increasingly accurate. Their virtual-navigation task enabled both egocentric (based on viewpoint matching and self-motion) and allocentric navigation via the rotation of the environment. Also, the landmarks gave positional or directional information to achieve the desired goal. The authors attempted to control for egocentric processing, which might aid children’s performance while making the task more challenging than in reorientation situations, which even children younger than five years can successfully solve. Newcombe (2019) assumes that children, even before the age of 2, use allocentric information in space (beacons, proximal and distal landmarks, boundaries) through their inertial navigation system. This ability then continuously develops until the age of 10, when it reaches the capacity to think relative to reference frames other than their own perspective.

According to a systematic review (Fernandez-Baizan et al., 2021), an early form of the allocentric strategy using coincident landmarks is apparent during the first year of life, yet children only become confident at the age of 6, and navigation performance continues to improve. This study defined allocentric navigation based on alignment to an external landmark. Based on using an anticipation paradigm conducted with infants in a room-size environment, Acredolo (1990) suggests that egocentric navigation dominated during the first 6 months, but this reference becomes less effective with the beginning of self-initiated locomotion of the infants. Around 11 months, this egocentrism can be overridden, but the spatial orientation still depends on the availability of landmarks in the environment for a significant portion of 16-month-old children, too. In the absence of landmarks, children at 6 to 11 months old switch back to an egocentric reference frame. The switching between egocentric and allocentric can be avoided, and all these findings can seamlessly be integrated with the trend of ‘egocentric first and allocentric later’ if we acknowledge that landmarks enabling PI are still part of the egocentric system, not allocentric.

Nardini et al. (2006) identified 3 types of reference frames in their study: 1. egocentric (body-centered) frame of reference: children orient in relation to their own bodies. This frame is useful when the body position remains constant between storage and recall. 2. environmental (room-centered) frame of reference: children orient themselves in relation to the fixed features of the room, such as the walls or furniture of the room. This includes egocentric information based on movement and an allocentric representation of space relative to landmarks. 3. internal (layout-centered) frame of reference: children orient to internal features of the layout, such as the relative positions of objects. The authors consider this to be a true viewpoint-independent representation. They claim that for 3-year-olds, the environmental reference predominates the egocentric, body-centered reference frame. In their experiment, the location of the test board in relation to the room characteristics influenced the youngest children’s responses more strongly than the location in relation to their own bodies. One explanation for this may be that 3-year-old children are able to mentally flip from the current view of the layout to a different view. However, Nardini et al. (2006) suggest that the children encode each location on an internal map that allows them to recall it from other perspectives. The viewpoint-independent layout-centered reference frame encompassing internal features of the spatial configurations develops only by the age of 5. Based on these facts, one could argue that there are multiple frames of reference that coexist between the ages of 3 and 5. This result is also consistent with the egocentric concept of PI because it enables the use of PI parallel in multiple sensory systems and combine their output based on egocentric motion-based and visual landmark-based reference frames within egocentric frameworks. The transition between egocentric and allocentric reference frames between the ages of 3 and 5 may be facilitated by external landmarks becoming increasingly reliable beacons relative to imprecise kinesthetic body-centered cues due to surplus visual information. However, visual tracking of multiple target objects in egocentric coordinates during the child’s movement may render the task too complex relative to the simplicity of tracking the navigator’s position relative to the static targets in an allocentric reference frame (Nardini et al., 2006; Clearfield, 2004).

The assumption that the egocentric spatial orientation develops earlier than the allocentric is supported by several neurophysiological findings, too. The sensory organ’s primary projection in the cerebral cortex reflects the sensory organ’s inherent coordinate system: the primary visual cortex is retinotopic, and the higher visual cortical areas are gaze-, head- and body-centered; hence they are all egocentric (Andersen et al., 1993). Besides the visual, other sensory inputs, including the inner ear vestibular system, spatial auditory, and the proprioceptive system, are part of the list of idiothetic cues that inform us about the change of the environment relative to our body (Asem and Fortin, 2017). Moreover, the frontal motor and somatosensory areas are also egocentric (Filimon, 2015). The longer maturation window of the hippocampal structures may also explain why children acquire the skill of effective allocentric representation over time (Bullens et al., 2010).

The confident use of the allocentric strategy may also relate to language development. Bell (2002) demonstrated that the development of spatial abilities is closely related not only to cognitive skills but also to linguistic skills. However, in another experiment by Nardini et al. (2006), 5-year-olds performed consistently above chance in a viewpoint-independent recall task despite their less matured spatial language skills. Van Hoogmoed et al. (2022) found that verbal working memory was independent of navigational performance, whereas visuo-spatial working memory was related to egocentric navigation. No such relationship was found with allocentric navigation.

It is likely that allocentric navigation is sensitive to the choice of external environmental factors in the given study and the familiarity of the task. For example, the previously presented experimental results were all conducted in spaces with different sizes (e.g., reaching spaces: Kaufman and Needham (1999); small spaces: Van Hoogmoed et al. (2022); Nardini et al. (2006); Acredolo (1990)), different visual accessibility and visual characteristics (vista spaces: Nardini et al. (2009), environmental spaces: Bullens et al. (2010)). Each of these factors can influence the reference frames used or preferred by the children (Li and Gleitman, 2002; Wolbers and Wiener, 2014). Among the factors rendering the interpretation of earlier experiments difficult is that children often had to suppress their own viewpoint by instruction, which imposed an enhanced cognitive load on the subjects and involved other cognitive components. Children have difficulties understanding the instruction, which requires them to adopt another viewpoint different from their own (Nardini et al., 2006). In this respect, experiments done in 3-dimensional virtual spaces are preferred over 2-D because they require less mental transformation to understand the task. On the other hand, virtual 3D spaces can be challenging for children because they lack the auditory and proprioceptive aspects of real environments. Based on the expanding methodological landscape, virtual navigation became a promising tool for testing navigation abilities in the preschooler population as it enables them to develop spatial representations of the environment. In addition, it opens up new methodological opportunities in the study of spatial navigation (Gabrielli et al., 2000).

### 5.1 The relationship of spatial navigation and the theory of mind

Whether the development of spatial navigation skills is independent of the maturation of other cognitive skills, and if not, then what the dependency between them is, has been a subject of long scientific discourse. Nardini et al. (2006) drew attention to the possibility that the widely documented late developmental onset of the viewpoint-independent reference frame (Piaget and Inhelder, 1967) can be explained by an extra task in those experiments: taking the viewpoint of someone else. Hence, the ability to switch reference frames may be contingent on the child’s status in acquiring the Theory of Mind (TOM) to take someone else’s perspective or vice versa. Therefore, the developmental milestones or deficits related to TOM may underlie the capacity for an earlier or later acquisition of allocentric reference frames. This seems to contradict the findings that children can reason about the mental states of others at a very early age. For example, Kovács et al. (2010) presented that infants seem to be able to attribute goals and intentions to others at a very early age and to maintain their beliefs in the absence of the agent. This seems to be an earlier ability than the emergence of allocentric strategies (around the preschool ages). However, many scientists drew attention to the limitations of research with children at a very young age (Rakoczy, 2012; Ruffman and Perner, 2005; Apperly and Butterfill, 2009; Poulin-Dubois et al., 2018).

Regarding the relationship between TOM and perspective-taking, we can delineate two approaches in the literature. According to the one (Aichhorn et al., 2006; Santiesteban et al., 2015; Keysar et al., 2003), TOM and visual perspective taking are somewhat or completely independent from one another. According to Aichhorn et al. (2006), our understanding of different reference frames relies only on certain aspects of TOM, e.g., on visual perspective taking, but it does not involve the behavior reasoning process as part of the typical TOM tests, such as the false-belief scenarios. In an experiment by Santiesteban et al. (2015) involving perspective taking, a camera (as an inanimate object) appeared to be the equivalent of a living person, suggesting that there is no need to attribute mental representations to an object to change the visual perspective.

According to the other approach, TOM and perspective-taking are related. On the one hand, the development of TOM may include the ability of visuospatial perspective-taking and cognitive perspective-taking (Howlin et al., 1999; Kloo et al., 2021), while spatial perspective taking may also assume the ability of mentalization. Schurz et al. (2013), in their meta-analysis, found common brain activation for false belief reasoning and visual perspective taking in the left dorsal temporoparietal junction (TPJ). Because the typical developmental window of acquisition of TOM between the third and the fifth year overlaps with the special period of sudden improvement in spatial navigation, spatial perspective taking, and the maturation of cognitive operations required by mentalization may interact positively during the development.

## 6 The spatial navigation ability of the elderly

Due to morphological changes in the key brain structures (hippocampus, frontal areas, and the retrosplenial cortex), the spatial orientation accuracy of adults exceeds those of the children and elderly, and both children and adult age groups prefer the egocentric strategy (Pullano and Foti, 2022). Although the egocentric preference and navigation skills are largely preserved with aging, spatial navigation in the elderly is increasingly dependent on the activity of the parietal lobe (i.e., precuneus, cuneus, inferior parietal lobe) (van der Ham and Claessen, 2020). Interestingly, despite the sustained egocentric skills, PI in the egocentric domain also becomes progressively difficult for the elderly (Colombo et al., 2017).

Elderly individuals often complain about spatial disorientation. According to research on the elderly, this sense of decline in orientation often transcends subjective experience (Burns, 1999). The association between these subjective feelings and the progressive deterioration of spatial navigation has been confirmed (Park et al., 2002; Moffat, 2009; Gazova et al., 2013; Colombo et al., 2017). Older participants require more time than younger individuals to create a cognitive map of the environment and make more errors when relying on cognitive maps for orientation (Iaria et al., 2009). Regarding gender differences in the age-related decline of spatial cognitive skills, Gazova et al. (2013) found no significant effects on egocentric and allocentric navigation. The decline in spatial navigation ability of the elderly has also been demonstrated by using virtual reality (Moffat and Resnick, 2002) as well as in real-world environments (Moffat, 2009). However, the environmental properties may also influence wayfinding strategies (Davis et al., 2023). Moffat (2009) reviewed the results of several tasks conducted in various real-world environments and found the performance of older adults inferior to that of younger adults in MWM analog tasks, as well as in scene-recognition, distance-ranking, route-execution, and map-placement tasks (Kirasic, 2000). While spatial abilities diminish across the lifespan, the visuospatial abilities underlying navigation performance in large-scale virtual environments appear independent of age (Muffato et al., 2016).

Another study focused on the effect of familiarity with the environment on navigation. Although spatial navigation performance declines with age in egocentric-based direction judgment, individuals perform better in familiar environments. However, this familiarity has no positive effect on allocentric-based proximity judgment. The authors suggest that familiarity enhances the ability to localize objects using egocentric strategies (Merriman et al., 2016).

Concerning neuroanatomy, a recent review by van der Ham and Claessen (2020), the mapping of brain areas involved in egocentric location knowledge is rather incomplete relative to that of the allocentric brain regions. In their systematic review, the measurements used by studies were categorized as follows: landmark tasks; localization tasks – egocentric and allocentric (mainly MWM); path knowledge tasks – route knowledge (e.g., route retracing) and survey knowledge (e.g., drawing a map). These domains relate to the following questions: What do we remember? Where do we localize? How do we get there? Their results showed that most research examines path recall, where age-related decline is observed in both route and survey cases. Gazova et al. (2013) compared the performance of older and younger individuals using analog MWM and, similar to previous studies, found that allocentric navigation is sensitive to aging. Brain imaging also confirmed these results. Antonova et al. (2009) showed decreased hippocampal and parahippocampal activity in elderly subjects relative to younger adults during a virtual version of the MWM. Beyond the methodological diversity of the studies, it can be stated that purely allocentric or egocentric navigation tasks are not available (Li and King, 2019). Claims regarding age-related deterioration in allocentric abilities should be critically examined as we have previously argued that tasks such as the MWM and the Triangle Completion can also involve egocentric computations (e.g., Wolbers and Wiener, 2014; Ekstrom et al., 2014). Thus, it is possible that these tasks may not have the sensitivity to dissociate the effect of age on PI and allocentric navigation. Particularly challenging is to determine the predominant reference frame when examining navigation during active movement in space, which requires continuous switching between egocentric and allocentric strategies (Coughlan et al., 2018; van der Ham and Claessen, 2020).

The types of reference frames (egocentric vs. allocentric) being used by the elderly are relevant for a range of other spatial navigation skills (Klencklen et al., 2012). For instance, the performance of the elderly in a spatial position recall task is just as good as that of younger adults as long as the task requires egocentric encoding (Pouliot and Gagnon, 2005). Therefore, when we address the age-dependent decline of cognitive and perceptive processes, it is important to clarify the role of reference frames as well. Uncovering which reference frame is more effective for older adults across different perspectives or task types can provide useful information for setting up navigation tools, as this would enable them to support navigation more effectively (Weisberg et al., 2024). Aside from the reference frames, elderly people with a decline in the allocentric navigation system may develop other strategies. For instance, analyzing body and gaze dynamics revealed a preference for geometry-based navigation in the elderly and landmark-based navigation in younger ones (Bécu et al., 2020).

To understand the nature of the age-dependent changes in spatial navigation, besides the cognitive factors, one must consider the sensorimotor aspects, too. The question is to what extent the decreased navigation skills can be explained by the degradation of spatial representations and by decreased visuomotor functions. Older people appear to rely more on proprioceptive cues in a virtual environment than younger ones (Lövdén et al., 2005). However, Moffat et al. (2006), while controlling for visuomotor factors, found a reduced activity of the hippocampus and the parahippocampal gyrus in the elderly. They also found a larger frontal lobe activity associated with environment encoding. All these changes were correlated with a decrease in the accuracy of spatial navigation. It appears that there is an age-related difference in step count and foot landing probability during navigation too. This highlights the importance of gait indicators. Indeed, the step count reflects whether someone is wandering in space, from which we can infer that they have a less effective cognitive map (Pawlaczyk et al., 2021).

Neuroimaging data corroborate behavioral findings in age-dependent decline in spatial cognition. Namely, both the volume of the hippocampus (Driscoll et al., 2003; Daugherty et al., 2016) and its activity during the spatial task is decreased in older adults as compared with young adults (Antonova et al., 2009; Konishi et al., 2013). Interestingly, neither the longitudinal studies (Korthauer et al., 2016; Daugherty and Raz, 2017) nor the experiments comparing the elderly with middle-aged groups (Korthauer et al., 2016) provided a satisfactory answer to the question of whether or not the atrophy of hippocampus can explain the spatial navigation deficit observed in elderly. The fact that epilepsy patients after a complete bilateral hippocampalectomy demonstrated spatial orientation performance non-inferior to normal control in navigation tasks as long as the task did not involve spatial memory (Teng and Squire, 1999) suggests that multiple brain regions are involved in spatial navigation, including the pariatal (Potegal, 1972; Cook and Kesner, 1988) and retrosplenial cortical areas (Takahashi et al., 1997; Wolbers and Büchel, 2005; Epstein and Higgins, 2007; Iaria et al., 2007; Auger and Maguire, 2013; Zhang and Ekstrom, 2013). In addition, age-dependent changes in other structures, such as the loss of volume in the caudate nucleus (Gunning-Dixon et al., 1998), may play an important role in the decline of spatial navigation with age (Colombo et al., 2017). One of the biggest caveats in this research area is the lack of well-designed longitudinal studies (Li and King, 2019).

Comprehensive neuroimaging studies on the elderly showing observable changes in prefrontal cortical areas suggest that the deficit of other cognitive functions may contribute to the spatial deficit (Shallice, 1982). Therefore, it is key to determine to what extent the decline in spatial orientation can be triggered by the deterioration of other cognitive functions, such as working memory (Iachini et al., 2009), executive functions (Shallice, 1982) and learning (Klencklen et al., 2012). Age-dependent changes in the working memory function of the dorsomedial prefrontal cortex may play an important role in the background of spatial cognitive decline by limiting the capacity to retain auxiliary information that might be critical for spatial memory performance (Iachini et al., 2009). Conversely, hippocampal dysfunction can also affect different aspects of cognitive functions: executive function (Papp et al., 2014), episodic and working memory (O'Shea et al., 2016), and PI skills (Yamamoto et al., 2014). Hence, the interaction between spatial memory and other cognitive functions is bidirectional.

Training involving route learning (e.g., recalling familiar routes, learning new routes, memorizing the spatial sequence of objects, recognizing landmarks or routes on a map) has a beneficial effect on spatial working memory, as it improves the encoding of sequential spatial information. This result is not only crucial for maintaining navigation abilities but also beneficial for everyday activities that require executive functions (Mitolo et al., 2017).

### 6.1 Dementia and spatial navigation

One of the most prevalent neurological diseases of our time is Alzheimer’s disease (AD). Broadening access to medical care prolongs the human lifetime and extends the expected lifespan of the population. Rising life expectancy combined with other factors such as declining fertility rates causes the ratio of the elderly to increase year by year, along with the prevalence of neurodegenerative diseases among older people and in general (Bloom and Luca, 2016). The prevalence of AD and mild dementia in the elderly around 60 years of age is 1%, while in the 85-year-old, it reaches 30% (Cummings, 2004). This trend, as well as the growing rate of GDP absorbed by medical care, highlights the importance of early prognosis of cognitive decline and developing interventions to relieve the symptoms.

One of the earliest and most specific symptoms of AD and vascular type of dementia is the spatial deficit, which causes the progressive decline of the spatial navigation skill, starting in the person’s larger environment and later invading the personal space and finally affecting everyday life (Moffat, 2009). In AD, there is an increased atrophy that MRI can detect in the area of the hippocampus and the amygdala when comparing it to the healthy elderly (den Heijer et al., 2006). The symptoms appear to correlate with the change of specific brain networks but not the severity of neurodegenerative changes (Tascone et al., 2017). However, neurodegenerative changes can also be observed in individuals who have not yet developed the disease but are at risk for AD (Kunz et al., 2015). The decline of PI when spatial cues are not available appears to be one of the earliest predictors of developing AD (Newton et al., 2024), particularly in individuals carrying the APOE ε4 allele (Kunz et al., 2015; Bierbrauer et al., 2020). The presumed cause is the molecular pathological changes occurring in the entorhinal cortex, such as Aβ and tau deposits. These protein deposits modify the function of grid cells, which play a role in allocentric PI (Howett et al., 2019; Newton et al., 2024). Additionally, they negatively affect the connection between the entorhinal cortex and the hippocampus (Karimani et al., 2024). Therefore, recognizing the earliest signs of spatial navigation decline is pivotal for the early diagnosis of the disease. The problem is the lack of normative databases for spatial navigation abilities, which could provide reference points for comparing early symptoms (Coughlan et al., 2018).

The expression of spatial navigation symptoms in both AD and Mild Cognitive Impairment (MCI) can be conceptualized by two models. According to the most common model, there is a visuo-perceptual deficit in the background. For example, the disruption of optic flow and the difficulty of coordinating gaze and visuospatial attention. The other model relates the symptoms to the cognitive map and the flexibility of the allocentric reference frame, including the ability to switch between egocentric and allocentric navigation, a function related to the retrosplenial cortex (Vlček and Laczó, 2014). Converging results, such as patients with AD perform worse in allocentric spatial navigation and tasks involving ordering places than healthy elderly control (Kalová et al., 2005; Hort et al., 2007), as well as they also express a preference for egocentric strategy, support the cognitive map model (Parizkova et al., 2018). In contrast, Tuena et al. (2021) reported deterioration of both types of navigation in Mild Cognitive Impairment.

In another study by Jheng and Pai (2009), patients in the early stage of dementia produced a reasonably good yet less elaborate cognitive map of familiar places than the healthy control, which suggests efficient egocentric and allocentric strategies. The authors argue that the early negative symptoms during spatial navigation may not be related to the integrity of cognitive maps. However, their contradictory result can be explained by the interpretational difficulty concerning the virtual MWM paradigm they used. Namely, it was difficult to dissociate the allocentric and egocentric strategies, regardless of whether the task was a relative location task (measured by the relative position of landmarks) or an absolute localization task (localization in a map).

Dementia is not only affecting spatial navigation, but it cooccurs with other symptoms, e.g., stress, anxiety, and making adaptation difficult (Marquardt, 2011). Living in a nursing home reduces physical activity (Scherder et al., 2010). This inactivity, combined with the lack of motivation and impaired smelling capacity (Ciciliati et al., 2021), causes weight loss and fragility, a leading and independent group of symptoms common in AD. It accelerates the progressive deterioration and, eventually, the likelihood of death (Chin et al., 1999). There are also speculations that spatial navigation dysfunction or visuospatial problems are responsible for the symptoms of aimless wandering. However, the etiology is poorly understood (Kwak et al., 2015).

## 7 Discussion

In this review, we argued that despite the extensive and deepening research in the field of spatial navigation, a few important but unjustified assumptions, ill-defined concepts, and methodological inconsistencies hinder the interpretation of results. Additionally, it is important to note that the usage of allocentric and egocentric terms is not always consistent.

Establishing a unified conceptual framework is challenging, as methodological diversity complicates the unified interpretation of different findings. We elucidated that the confusion of PI with allocentric reference frames generated contradictions in the literature. Furthermore, we argued that regarding non-human research results, using the “shortcuts” as evidence for cognitive maps is incorrect, as shortcuts can be computed from PI without a cognitive map. Therefore, we propose that future research should take into account that landmark-aligned optimal behavior may not automatically be evidence of a cognitive map.

It remains a challenging question, nevertheless, how children’s spatial cognitive development is embedded in the context of evolving executive functions and general cognitive skills. There are significant stepwise changes in multiple cognitive domains during the period between 3 to 5 years, which makes it very tempting to relate the adoption of TOM to the acquisition of allocentric reference frames. However, the allocentric reference frame does not replace egocentric; instead, they coexist. Our concept is in line with Piaget’s developmental model, according to which the egocentric viewpoint precedes the viewpoint-independent allocentric representation mode, which is more vulnerable to age-related changes and sensitive to environmental and experimental factors. These findings are concordant with results indicating that allocentric orientation is more susceptible to cognitive decline (Kalová et al., 2005; Hort et al., 2007; Parizkova et al., 2018). Despite its vulnerability, the allocentric reference frame offers advantages requiring less cognitive load and neuronal computation during movement than the egocentric mode.

We argued that the spatial reference frames provide a very sensitive tool to study age-dependent changes in spatial cognition. This conceptual device could serve as an important early diagnostic tool for the most devastating neurodegeneration diseases, such as AD, because they affect the same brain areas as the ones involved in spatial navigation and spatial awareness. The demand for such tools is imminent. Therefore, it is necessary to critically overview the methodological landscape from time to time and synthesize the results in a coherent narrative to attain conceptual clarity and point attention to new research directions for future professionals.
